# Supplementary material for: Systematic optimization of Cas12a base editors in wheat and maize using the ITER platform
Source: Genome Biol. 2023 Jan 13;24:6. doi: 10.1186/s13059-022-02836-2 (PMC9838060; doi:10.1186/s13059-022-02836-2)
Supplement: Supplementary file 1 — Additional file 1: Fig. S1. Establishment of high content analysis pipeline. Fig.S2. ITER validation for Cas9 base editors using GFP reporters. Fig. S3. ITER validation for Cas9 base editors at endogenous sites. Fig. S4. Iterative optimization of Cas12a-ABEs using ITER. Fig. S5. Cas12a-ABEs efficiency at endogenous sites in protoplasts. Fig. S6. Cas12a-ABE efficiency in stable wheat transformants. Fig. S7. Cas12a-CBEs and LbCas12a efficiency at endogenous sites in protoplasts. Appendix. Raw gel pictures. [file 13059_2022_2836_MOESM1_ESM.docx]

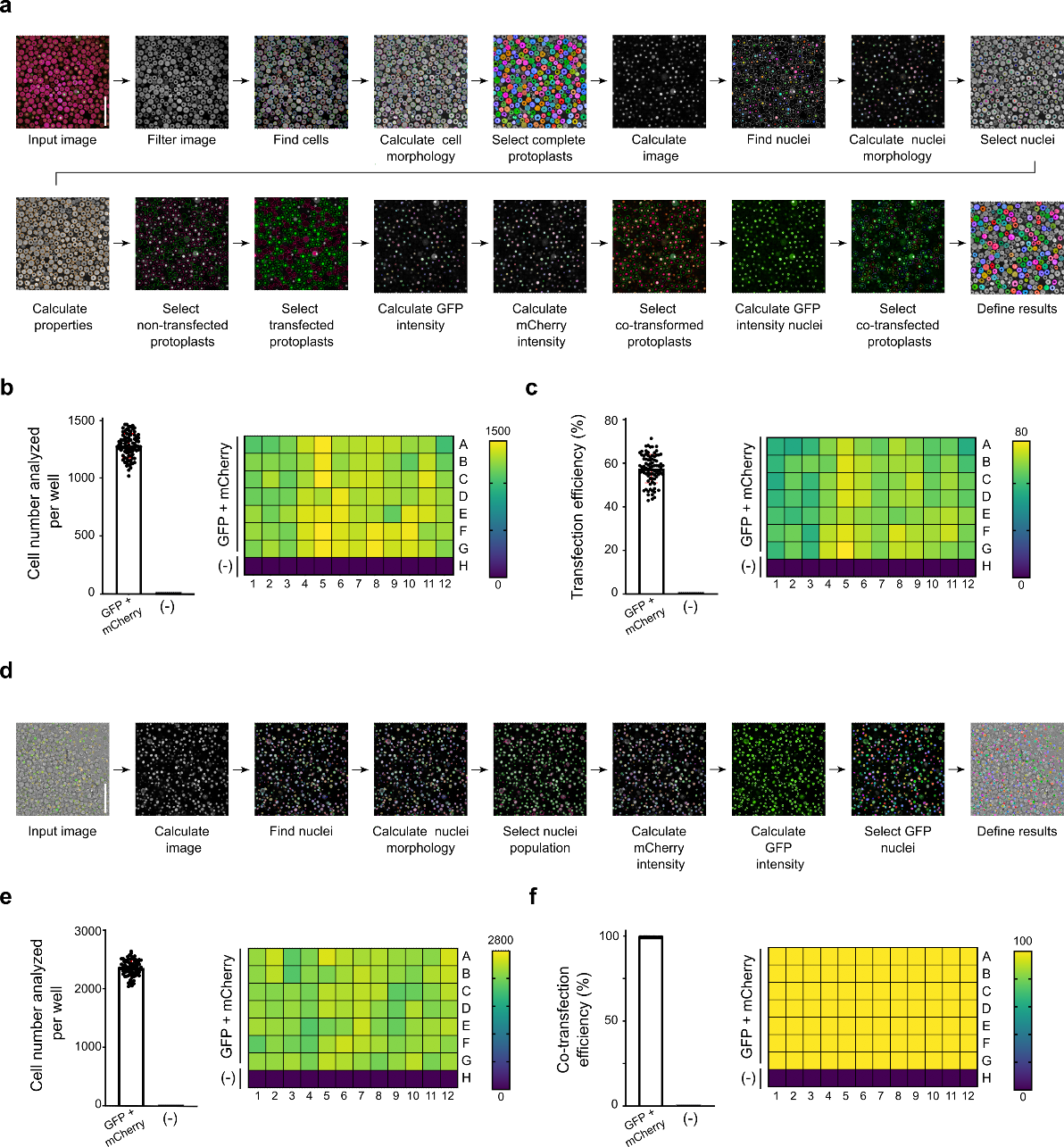


**Fig. S1: Establishment of high content analysis pipeline.**

**a,** Representative images from image segmentation and quantification workflow for wheat protoplasts on the OPERA® High Content Screening System. **b,** Cell number at individual wells after protoplast transfection as measured by the total number of mCherry positive wheat cells. **c,** Transfection efficiency at individual wells as measured by mCherry positive wheat cells over the total number of segmented cells. **d,** Representative images from image segmentation and quantification workflow for maize protoplasts on high content imaging platform. **e,** Cell number at individual wells after protoplast transfection as measured by the total number of mCherry positive maize cells. **f,** Co-transfection efficiency as measured by the number of GFP over the number of mCherry positive cells in maize. Negative controls (-) do not contain vector DNA (b,c,e,f). The grid shows the 96-well plate positions and gradient color coding represent number of cell analyzed (b,e), transfection efficiencies (c) or co-transfection efficiency (f). Scale bars in a,d: 200 µm


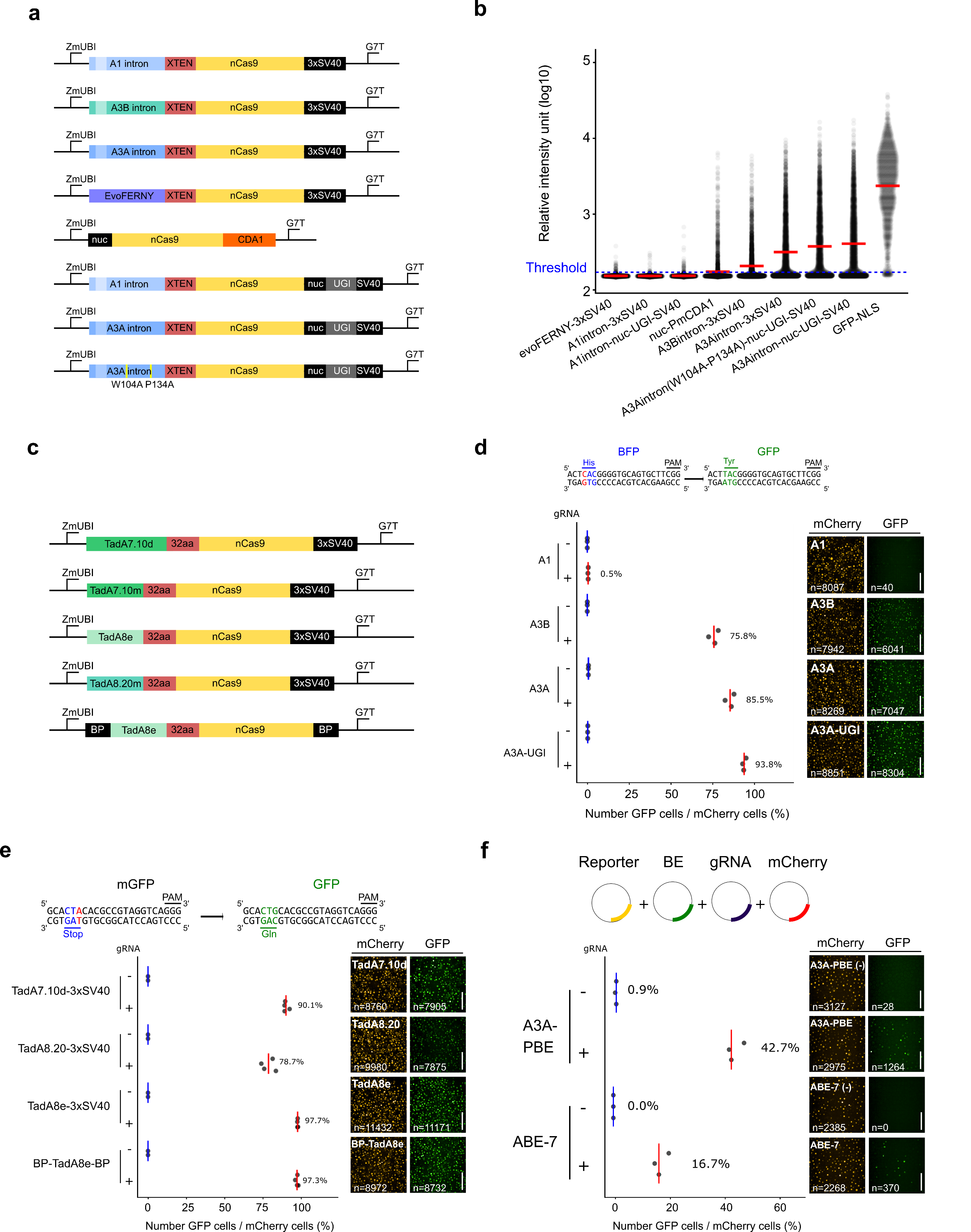


**Fig. S2: ITER validation for Cas9 base editors using GFP reporters**

**a,** Schematic representation of Cas9-CBE architectures tested in this study. **b,** Relative GFP intensity of individual cells transfected with nCas9 CBEs. The GFP-NLS positive control was transfected with pZmUBI:GFP-NLS. Red bars represent mean. **c**, Schematic representation of Cas9-ABE architectures tested in this study. **d-e,** Base editing efficiencies for Cas9-CBEs (d) and Cas9-ABEs (e) as measured by the rate GFP conversion in maize cells. **f,** Base editing efficiency for A3A-PBE [33] and ABE-7 [34] as determined by the rate of GFP conversion in wheat cells. Editing rates were calculated from three or four independent biological replicates that are depicted as dots. Red and blue lines represent mean efficiencies with or without gRNAs, respectively. Representative pictures of protoplasts showing mCherry or GFP fluorescence. n indicates the total number of cells measured after image segmentation in the mCherry and the GFP channel (d-f). Scale bars in d-f: 200 µm

**
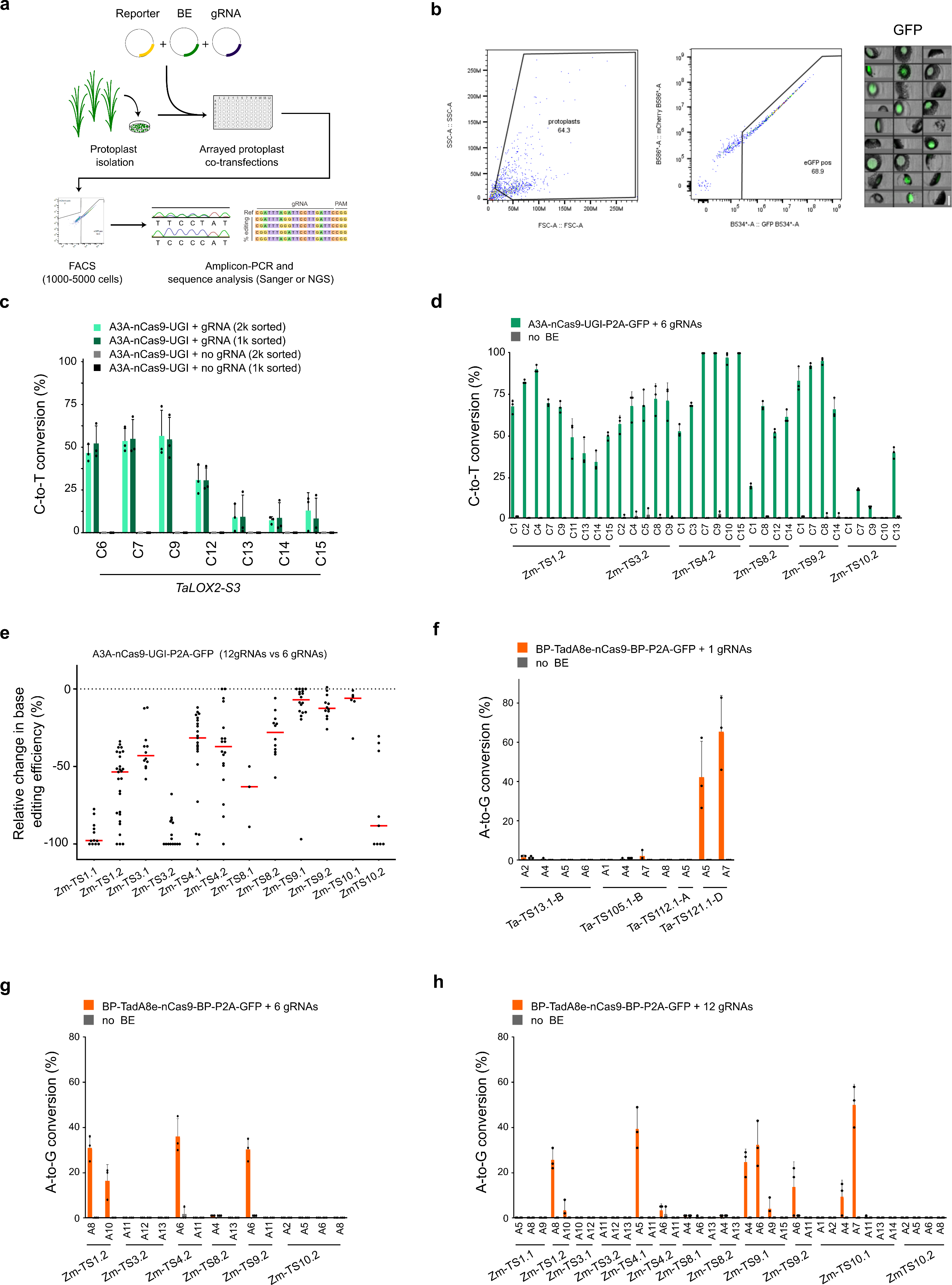
**

**Fig. S3: ITER validation for Cas9 base editors at endogenous sites.**

**a,** Schematic representation of Fluorescence-Activated Cell Sorting (FACS) and genotyping workflows. Vectors are co-transfected in protoplasts and sorted on GFP signal directly in dilution buffer. Solution containing lysed protoplasts is used as template for PCR and resulting amplicons are used for Sanger sequencing or NGS. **b,** Gating strategy used for sorting protoplasts expressing GFP. Pictures show intact wheat cells expressing nuclear GFP after sorting. Scale bar**:** 20 µm. **c,** Simplex base editing efficiency of A3A-nCas9-UGI at *TaLOX2-S3* (c) as measured by Sanger sequencing. One wheat homeolog was analyzed. Transfection of the CBE without the gRNA was used as a negative control. **d,** Multiplex base editing efficiency of A3A-nCas9-UGI at six maize targets. Percentages indicate the proportion of C-to-T base conversion. Transfection of the CBE without the gRNA was used as a negative control. The x-axis indicates targeted Cytosines at different positions along the protospacer, with the PAM-distal base being position 1 (c-e). **e,** Relative change in multiplex base editing efficiency between 6 and 12 gRNAs in maize protoplasts. **f,** Simplex base editing efficiency of BP-TadA8e-nCas9-BP at four wheat targets. **g-h,** Multiplex base editing of BP-TadA8e-nCas9-BP at 6 (g) or 12 (h) maize targets. The x-axis indicates targeted Adenine at different positions along the protospacer, with the PAM-distal base being position 1 (g-i). Editing rates were calculated from three independent biological replicates that are depicted as dots (c-i). Transfection of the cells with a GFP expressing vector (no BE) was used as a negative control (e-i).


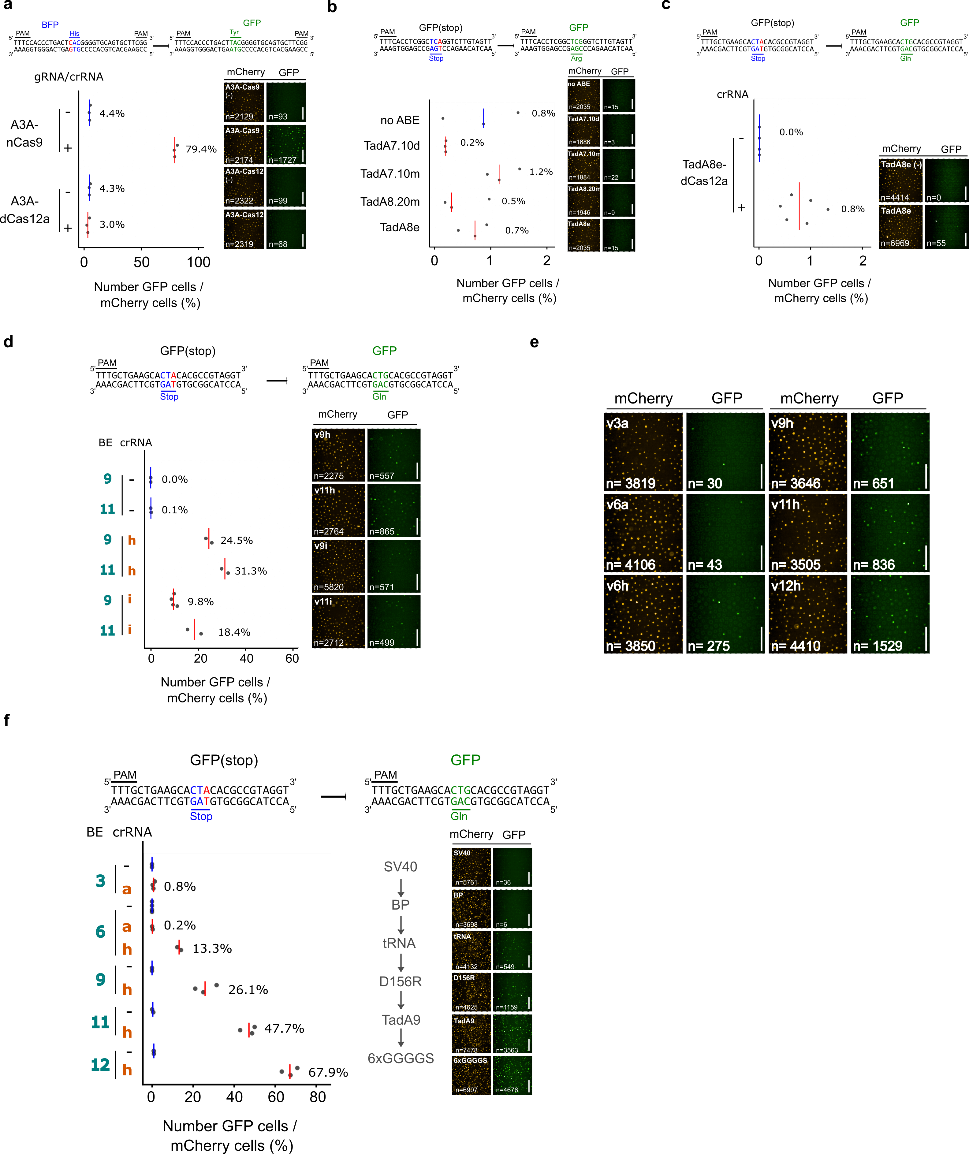


**Fig. S4: Iterative optimization of Cas12a-ABEs using ITER**

**a,** Base editing efficiencies for A3A-nCas9 or A3A-dCas12a in wheat as measured by the rate of BFP to GFP conversion. **b,** Base editing efficiencies for Cas12a-ABEs as measured by the rate of GFP recovery (stop to Arg) in wheat cells. **c,** Base editing efficiency for TadA8e-dCas12a as measured by the rate of GFP recovery (stop to Gln) in wheat cells. **d,** Base editing efficiency for Cas12a-ABEs as measured by the rate of GFP recovery (stop to Gln) in wheat cells. **e,** Representative pictures showing mCherry and GFP positive cells measured during the comparative analysis of key Cas12a-BE architectures in Fig2. c’’. **f,** Comparative analysis of key Cas12a-ABE architectures obtained along the optimization path as measured by the rate of GFP recovery (from stop to Gln) in maize cells. Components leading to increased activity are shown at the right of the panel. Representative pictures show fluorescence measured for mCherry and GFP cells and the number of cells analyzed. Editing rates were calculated from two to four independent biological replicates that are depicted as dots. Scale bars in a-f: 200 µm

**

**

**Fig. S5: Cas12a-ABEs efficiency at endogenous sites in protoplasts**

**a-b,** Base editing efficiencies of TadA8e-dCas12a (v9h) in simplex (a) or multiplex (b) as measured by the proportion of NGS reads converted from A-to-G at six wheat targets. **c-d,**  Base editing efficiencies of six Cas12a-ABE configurations in simplex or multiplex at four wheat off-targets located on homoeologous genes as measured by the proportion of NGS reads converted from A-to-G. DNA sequences on the top row are the on-targets, red bases indicate mismatches with the crRNA at off-target homeologs. **e,** Indel rate measured at wheat on-targets for six Cas12a-ABE configurations. Color code for Cas12a-ABE architectures is same as (c). **f,** Simplex Base editing efficiencies of TadA8e-dCas12a (v9h) in maize as measured by the proportion of reads converted from A-to-G at four targets. Editing rates were calculated from two or three independent biological replicates.


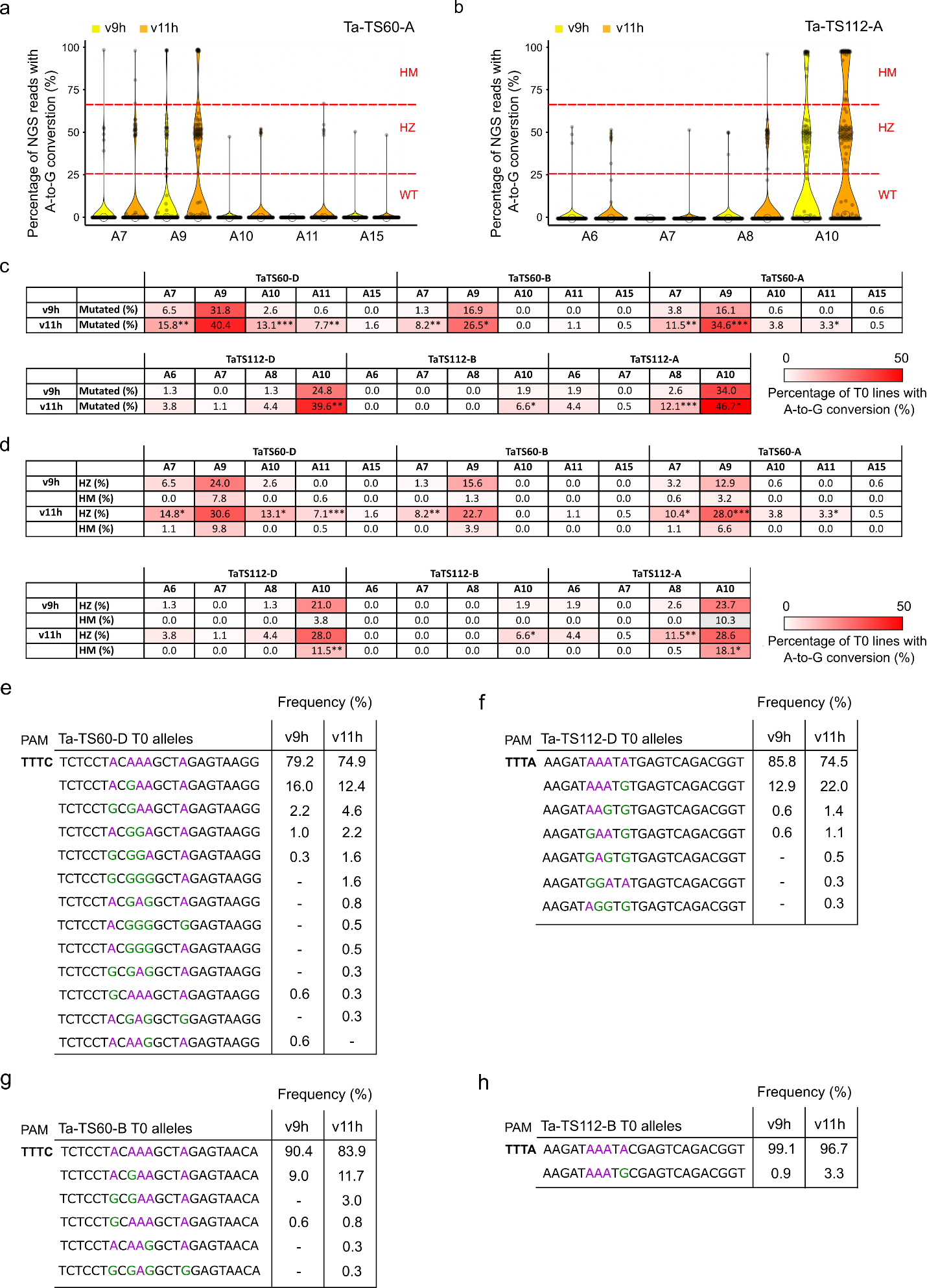


**Fig. S6: Cas12a-ABE efficiency in stable wheat transformants**

**a-b,** Base editing efficiencies in T0 wheat plants at TS60-A (a) and TS112-A (b) as measured by the proportion of NGS reads converted from A-to-G. Red dashed lines defines 3 classes of genotypes: wild type plants for samples with less than 25% of edited reads, heterozygous for 25-70% and homozygous for more than 70%. **c-d,** Frequency of individual T0 wheat plants carrying heterozygous or homozygous A-to-G conversion at individual positions of the targets. Combined heterozygous (HZ) and homozygous (HM) frequencies are shown in **c**, individual frequencies in **d**. Values are depicted for TaTS60 and TaTS112 at the three subgenomes (n=154-157 for v9h and n=182-183 for v11h). Asterisks in (**c**-**d**) represent significant difference in efficiency between v9h and v11h as measured by Z-score test for two proportions (*: p<0.05; **: p<0.01; ***: p<0.001). **e-h,** Allelic frequency in base edited T0 wheat plants at TS60-D (e), TS60-B (f), TS112-D (g) and TS112-B (h) (TS60: v9h: n=312 alleles; v11h: n=366-368 alleles; TS112: v9h: n=316-318 alleles; v11h: n=368 alleles). Base edited nucleotides are marked in green.

**

**

**Fig. S7: Cas12a-CBEs and LbCas12a efficiency at endogenous sites in protoplasts.**

**a,**  Base editing efficiencies of six Cas12a-CBE configurations in multiplex at four wheat off-targets located on homoeologous genes. Efficiencies are measured by the proportion of reads converted from C-to-T. DNA sequences on the top row are the on-targets, red bases indicate mismatches with the crRNA at off-target homeologs. Asterisks depicts significant difference in efficiency as calculated by Kruskal-Wallis test followed by Dunn post-hoc test (*: p<0.05). **b,** Multiplex indel rate of LbCas12a at eight wheat off-targets located on wheat homeologous genes. DNA sequences on the top row are the on-targets, red bases indicate mismatches with the crRNA at off-target homeologs. Violin plots represent pooled indel rates at all targets for individual LbCas12a architectures. Significance is calculated by two-way ANOVA test with Tukey HSD post-hoc test at p<0.05. Editing rates were calculated from three independent biological replicates that are depicted as dots on barplots. **c-d,** Pattern of indels generated by LbCas12a at four wheat (c) or maize (d) on-targets. Insertions and deletion from 1 to >19 base pairs are shown.

**Appendix**

Raw gel pictures during amplicon isolation. Red rectangles depict PCR bands that were isolated and analyzed. Individual gels correspond to figure panels indicated on top left corner. Name of the target size and expected PCR product sizes are indicated on gel pictures.

**
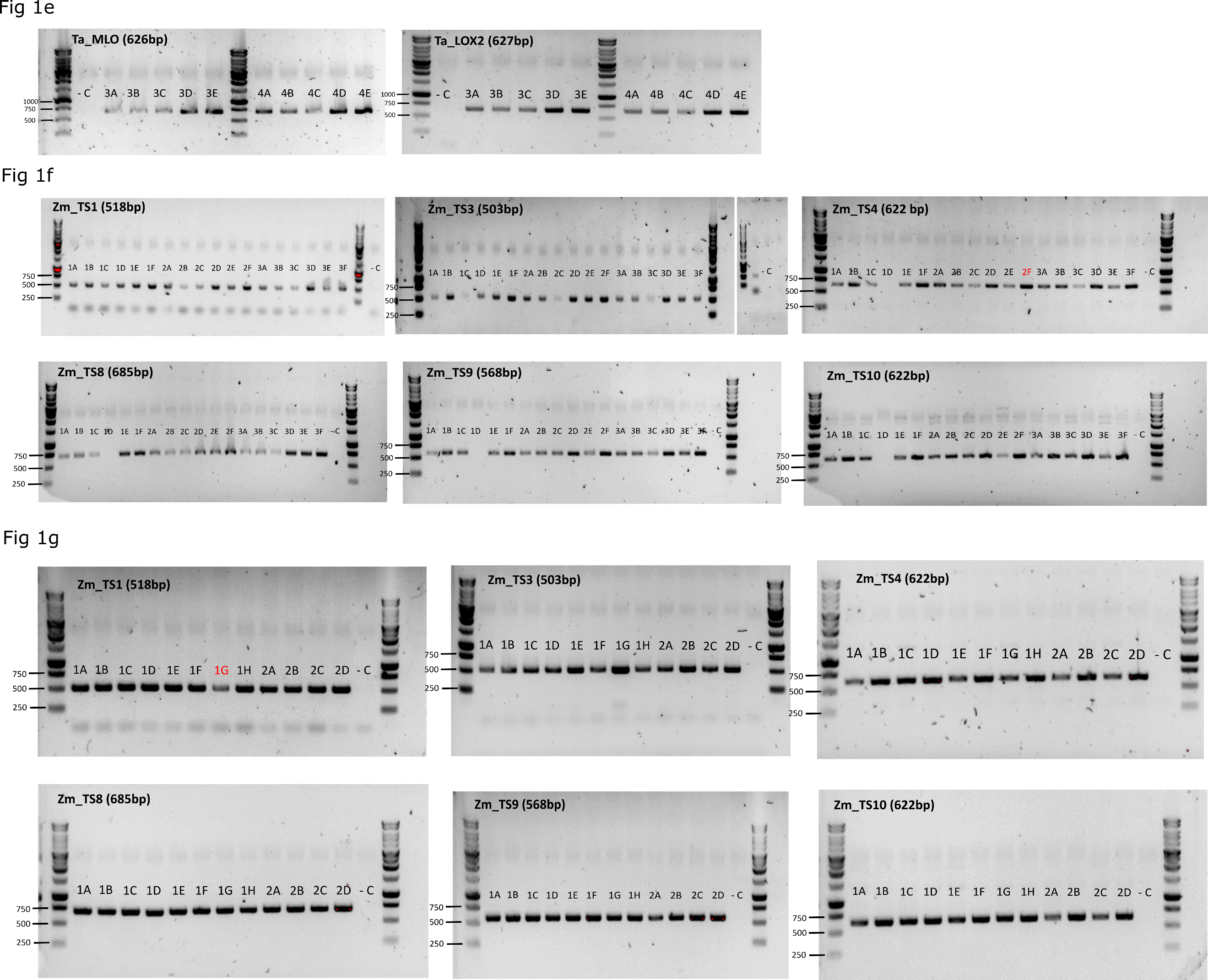
**

**
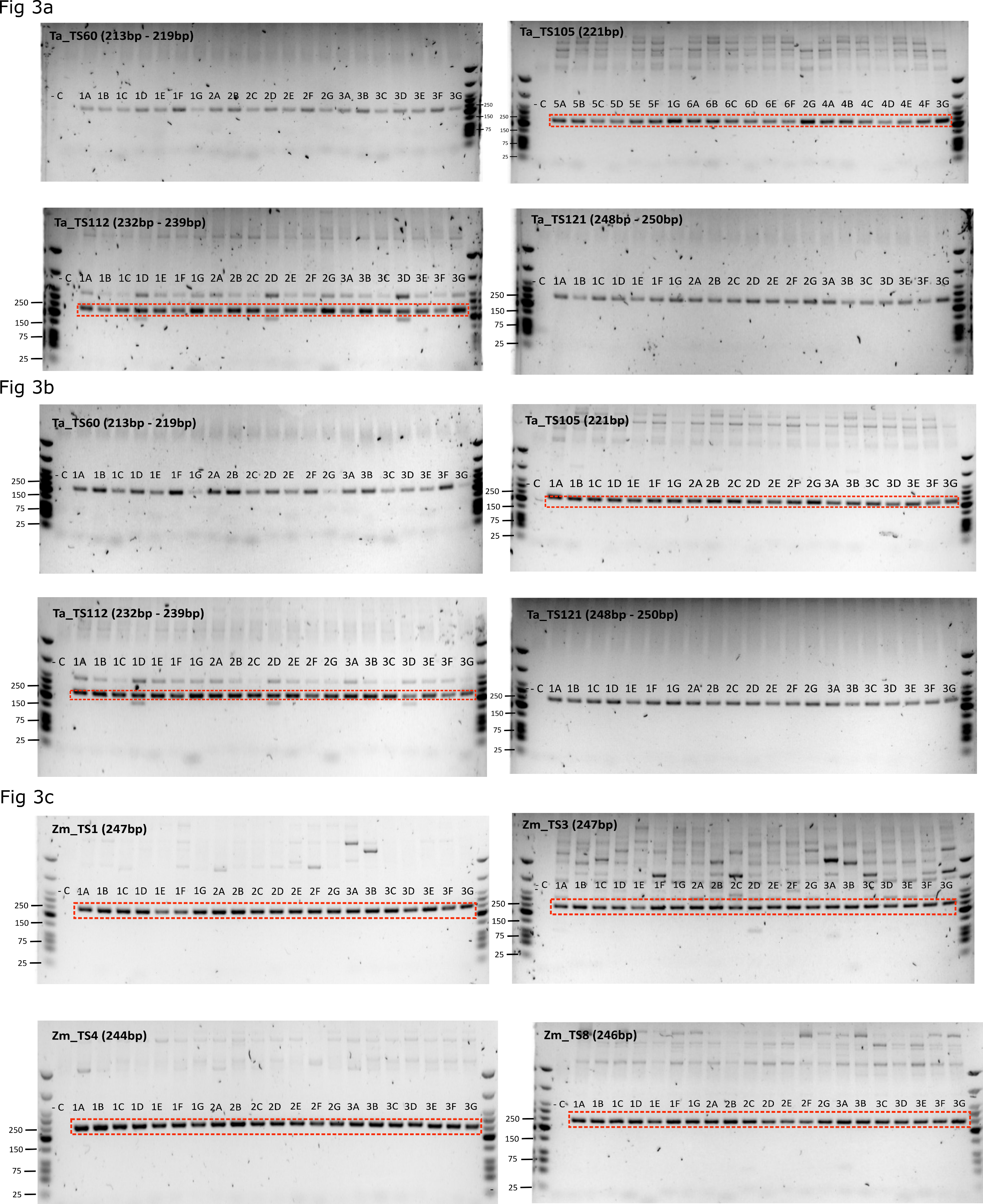
**

**
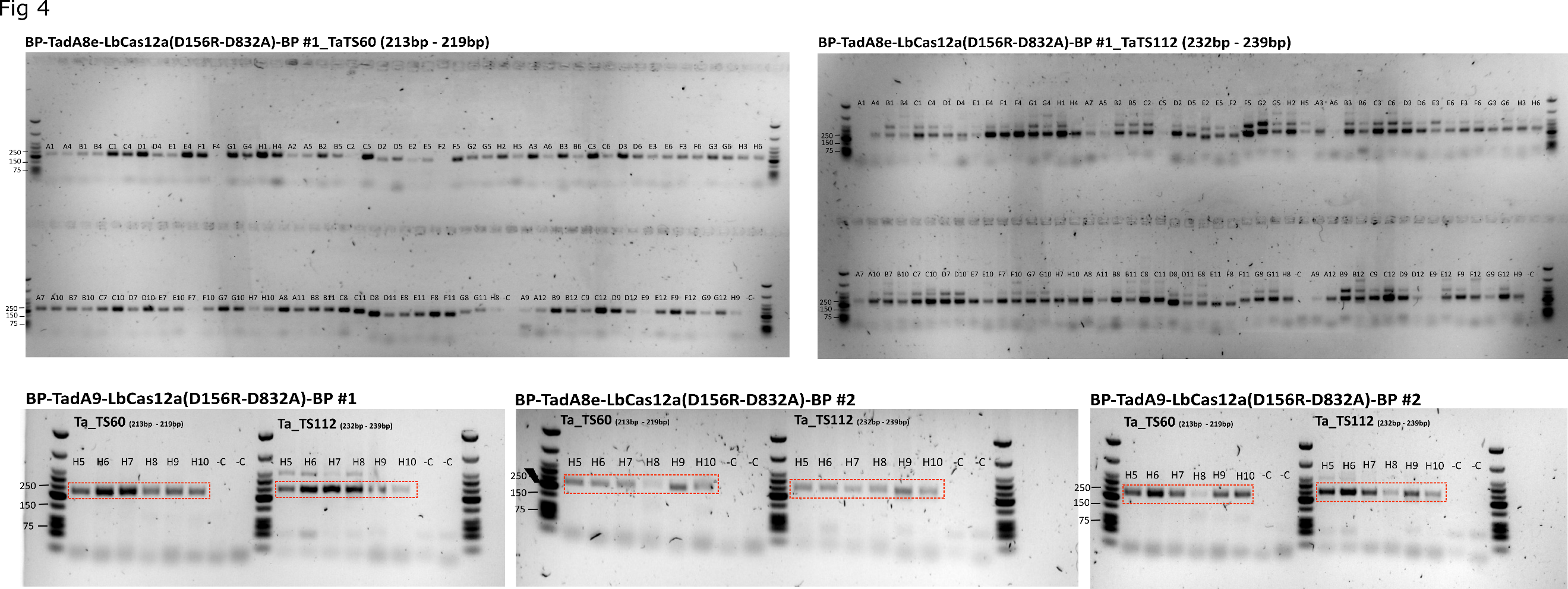
**

**
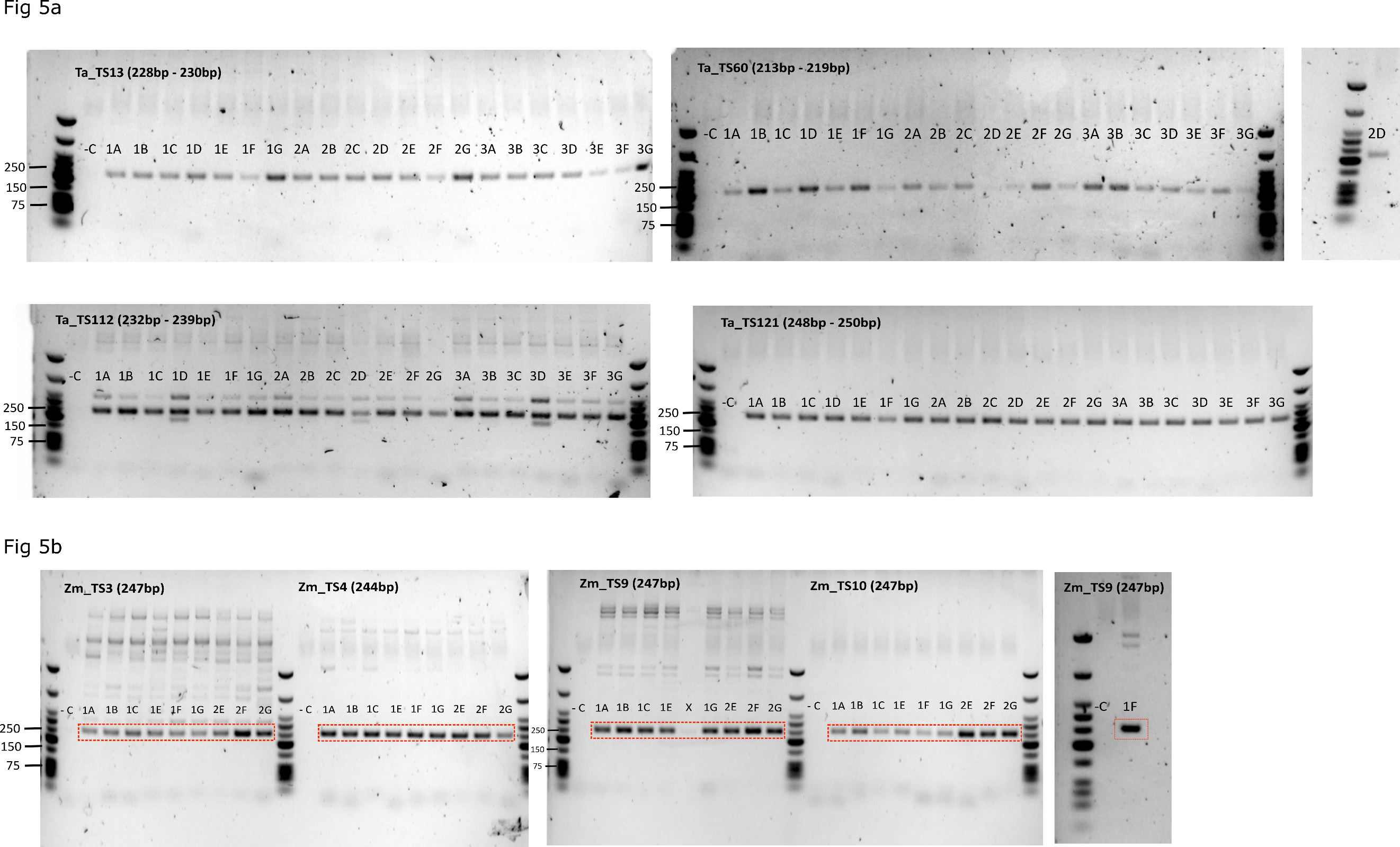
**

**
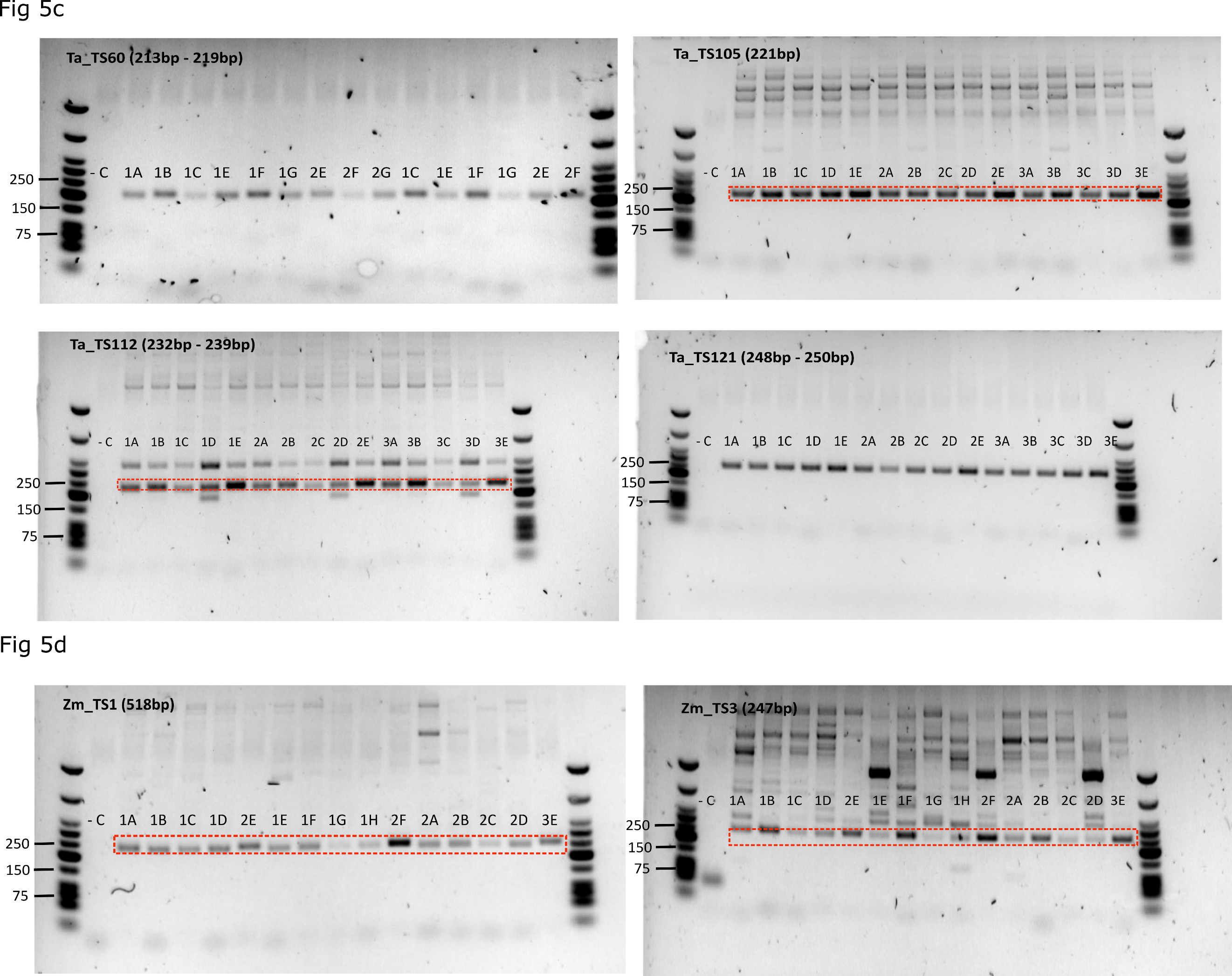
**

**
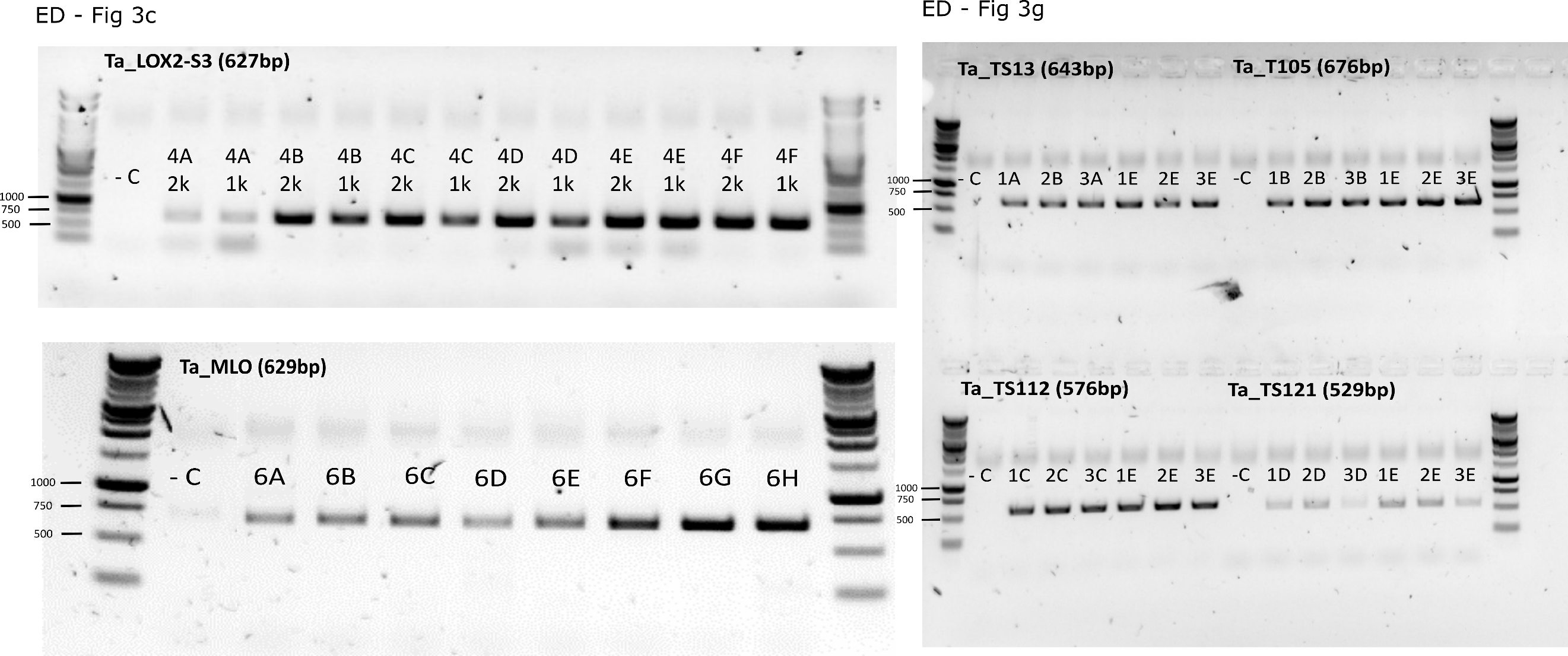
**

**
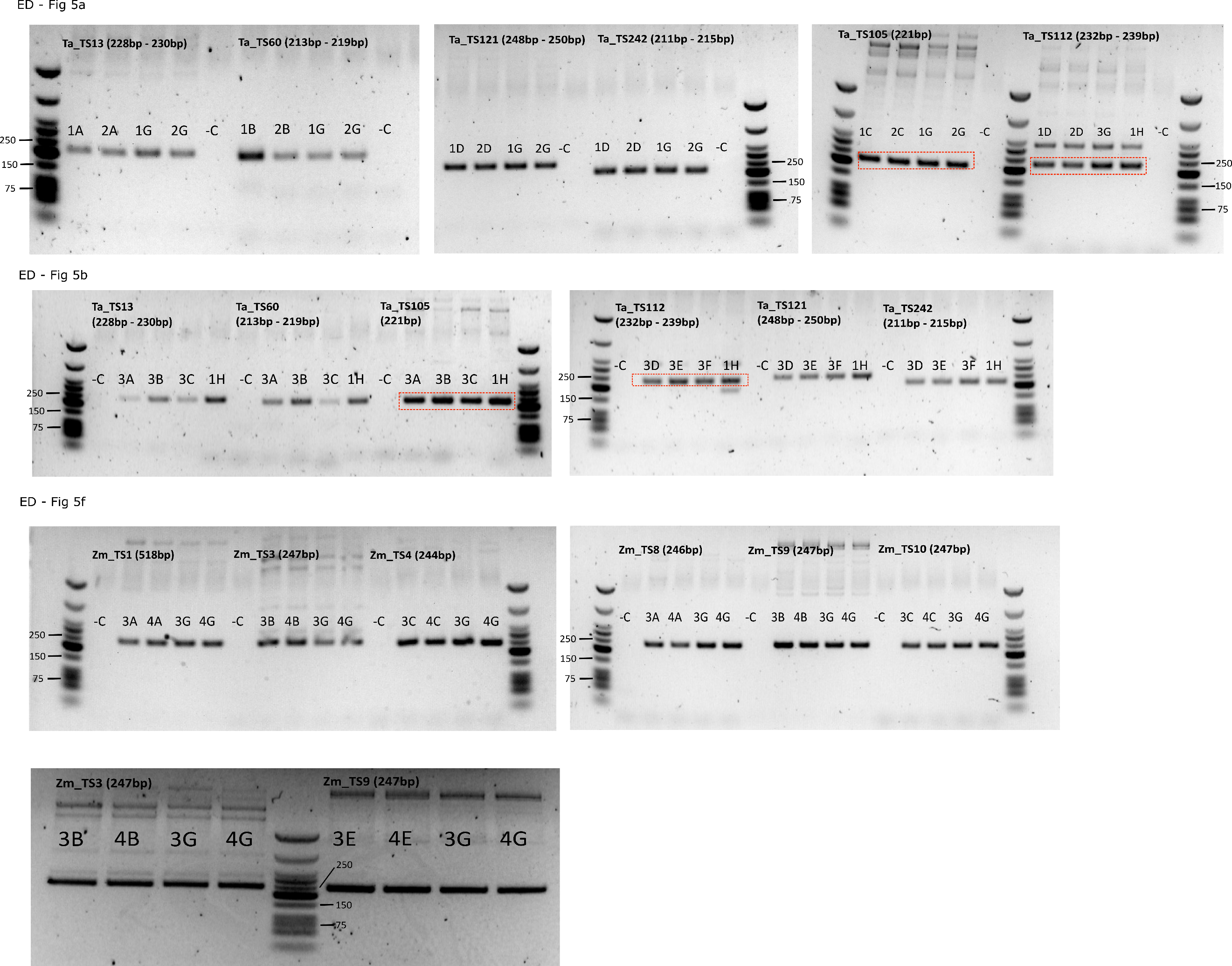
**
