# Supplementary material for: Systematic optimization of Cas12a base editors in wheat and maize using the ITER platform
Source: Genome Biol. 2023 Jan 13;24:6. doi: 10.1186/s13059-022-02836-2 (PMC9838060; doi:10.1186/s13059-022-02836-2)
Supplement: Supplementary file 10 — Additional file 10. Review history. Comments and statistical assessment from reviewer 3 are missing in the first round of revision. [file 13059_2022_2836_MOESM10_ESM.docx]

Review History

**First round of review**

**Reviewer 1**

**Were you able to assess all statistics in the manuscript, including the appropriateness of statistical tests used?**

Yes, and I have assessed the statistics in my report.

**Comments to author:**

Base mutations in organisms are important resources for functional phenotypes. Thus, base editors (BE) that introduce single or oligomer base substitutions are important technological directions in genome editing. At present, a set of base editors based on nCas9 or dCas9 had been developed in animals and plants. BE based on Cas12a had not been well established. The presented manuscript described a work using Iterative Testing of Editing Agents (ITER) strategy that allowed to test editing agents up to 96 combinations in protoplasts. From my personal point of view, this design can indeed screen the elements related to gene editing technology with high throughput, and find the combination of elements with good activities. The presented data using NGS also supported that Cas12 based ABE along with CBE showed good and significantly improved mutagenesis rates. The weakness of this study should be that it lacks a solid in planta data or well-organized data (Fig.4) to support the ultimate meaning of this study, which we can find a system work well in vivo to get inheritable mutations and therefore could be used into genetic improvement. I personally suggested that it could be acceptable for publication after this major concern could be well addressed or providing the supplemental data.

Major concerns and suggestions:

In Fig.4; Fig.3 and Fig. S6. For ITER strategy, the CRISPR/Cas effectors should be split as the Cas12a-ABE (Fig. 2a), crRNA (b) and artificial target of dGFP et al (c). They should be simultaneously and transient transformed into protoplasts. These in vitro system does have advantage of efficiency to test in cells. But the in vivo system working in cell nucleus in vivo is different and much more complicated. As mentioned above, this work could be strongly supported here by data such as obtaining a mutation with a well-defined target and achieving a genetically improved phenotype. Well-organized data could be: in vivo Cas12a-ABE target, vectors, transformants verification or molecular features of ABE, on target mutation efficiency, heredity of mutations; mutant phenotypes. I suggested that the plant of claimed homologous with target phenotype could be shown.

Other specifics:

Introduction section line 59-64 and References 13-18 and Fig.1 & Fig.2

The current text listed the previous researches of the Cas12 system derived BE technologies (line 59-64). I think it should be elaborated more detail here to support your ITER design. All elements used in ITER had been tried by other, the strength of this study should be more combinations were efficiently tested. Am I right? Please clarify it in text.

I think that deaminase itself can introduce mutations that may restore dead-GFP reporters et al. and off-target effect. Unlike human medical research, off-target mutations in plants can be ignored. Using well established control, and using the data associated with PAM and targeting window, the data on mutation efficiencies are meaningful. Off-target data could be provided as supplementary data and move this section into discussion.

**Reviewer 2**

**Were you able to assess all statistics in the manuscript, including the appropriateness of statistical tests used?**

No, I do not feel adequately qualified to assess the statistics.

**Comments to author:**

The manuscript of Gaillochet et al. describes the experiments to systematically optimize base editors for use in plants which the authors refer to as ITER. ITER is based on the transfection of 96-welled arrayed wheat and Corn protoplast. Every round of optimization can be achieved within three weeks which allows at fast pace enzyme optimization, which in combination with the high through-put approach is exceptional for plants.

The aim of the study was to optimize Cas12a based base editors. As a matter of fact, Cas12 base editor are less efficient than Cas9 base editors as they are based on a nuclease-dead and not on nickase-proficient DNA binding moiety. The authors tested different components of the enzyme for improvements namely the kind of editor, the crRNA, linker, NLS and mutations in LbCas12. The importance of all these factors was known form a row of published studies.

The authors succeeded in improving Cas12a-editors significantly from back ground noise to up to 20% editing at genomic sites in the best case. They also were able to document mutation induction in regenerated T0 plants at rates from 3 to almost 50 %

This is definitely an interesting piece of work. However; I want to raise two concerns:

What I missed was the documentation that the induced mutations were indeed transferred as expected to the T1 generation

What I found a worrisome ss that optimized editor induced beside the on-site mutation also quite efficiently off-site mutations at related sites. Have the author any evidence that the editor is not also inducing edits independent of the sequence in a genome wide manner?

**Authors Response**

**Point-by-point responses to the reviewers’ comments:**

Reviewer #1:

Base mutations in organisms are important resources for functional phenotypes. Thus, base editors (BE) that introduce single or oligomer base substitutions are important technological directions in genome editing. At present, a set of base editors based on nCas9 or dCas9 had been developed in animals and plants. BE based on Cas12a had not been well established. The presented manuscript described a work using Iterative Testing of Editing Agents (ITER) strategy that allowed to test editing agents up to 96 combinations in protoplasts. From my personal point of view, this design can indeed screen the elements related to gene editing technology with high throughput, and find the combination of elements with good activities. The presented data using NGS also supported that Cas12 based ABE along with CBE showed good and significantly improved mutagenesis rates.

The weakness of this study should be that it lacks a solid in planta data or well-organized data (Fig.4) to support the ultimate meaning of this study, which we can find a system work well in vivo to get inheritable mutations and therefore could be used into genetic improvement. I personally suggested that it could be acceptable for publication after this major concern could be well addressed or providing the supplemental data.

*Response: In response to the reviewer’s request and those made by R2, we have added additional experimental data demonstrating the inheritance of edits in T1 (next generation) wheat plants lacking the base editing vector (null segregants). This enables us to conclude that the base edited alleles are transmitted by the T0 and can be used for genetic improvement. Of the 24 T1 lines genotyped (n=94 plants), 22 contained inherited alleles. As suggested by R1, we have updated figure 4 and supplemental figure 6 with this new data and modified our original representations of the T0 data.*

Major concerns and suggestions:

- In Fig.4; Fig.3 and Fig. S6. For ITER strategy, the CRISPR/Cas effectors should be split as the Cas12a-ABE (Fig. 2a), crRNA (b) and artificial target of dGFP et al (c). They should be simultaneously and transient transformed into protoplasts.

*Response: We are not entirely sure what is being requested here. We utilize two methods to deliver the plasmids to plant cells: 1, individual plasmids for Cas and guide RNAs transfected into protoplasts via PEG (Schematic on additional file 1, FigS3a) and 2, biolistic delivery of the same plasmids into wheat embryos (Schematic on Fig. 4a). As illustrated in supplemental figure 1, transfection of two reporter plasmids into protoplasts is simultaneous.*

These in vitro system does have advantage of efficiency to test in cells. But the in vivo system working in cell nucleus in vivo is different and much more complicated. As mentioned above, this work could be strongly supported here by data such as obtaining a mutation with a well-defined target and achieving a genetically improved phenotype. Well-organized data could be: in vivo Cas12a-ABE target, vectors, transformants verification or molecular features of ABE, on target mutation efficiency, heredity of mutations; mutant phenotypes. I suggested that the plant of claimed homologous with target phenotype could be shown.

*Response: The in vitro methods we use take place in the plant cell nucleus. We use a GFP-reporter strategy to initially screen for optimal vector configurations, then use several endogenous genes to confirm the reporter results in protoplasts and in vivo (stable wheat plants). The new genotyping results in advanced generations show the inheritance of diverse heterozygous and homozygous alleles. Taken together, our approach addresses all points raised by the reviewers to validate base editing activity. We did not select targets to engineer traits as we focused on the production of DNA base changes which is the primary objective of base editors, but this will certainly be of interest in follow up studies.*

Other specifics:

- Introduction section line 59-64 and References 13-18 and Fig.1 & Fig.2

The current text listed the previous researches of the Cas12 system derived BE technologies (line 59-64). I think it should be elaborated more detail here to support your ITER design. All elements used in ITER had been tried by other, the strength of this study should be more combinations were efficiently tested. Am I right? Please clarify it in text.

*Response: Good point, we now reference the original studies that have published the variants used in our study (lines 65-69) and clarified that their use for optimizing Cas12a-BE remains unexplored in plants.*

*We have added the following text: “Cas9-BEs have been optimized by individually modifying components such as the nuclear localization signal (NLS;[23]), deaminase [14,24,25]), and linker [21]. Cas12a in turn has been optimized with different nuclease variants [13,26,27] and modifying the crRNA scaffold [28]. However, it is unclear whether any of these improvements, or combinations thereof, would improve Cas12a-BE efficiency.*

I think that deaminase itself can introduce mutations that may restore dead-GFP reporters et al. and off-target effect. Unlike human medical research, off-target mutations in plants can be ignored. Using well established control, and using the data associated with PAM and targeting window, the data on mutation efficiencies are meaningful. Off-target data could be provided as supplementary data and move this section into discussion.

*Response: We have provided off-target data in the additional file 1 and added a paragraph in the discussion starting at line 431 as suggested by the reviewer. We indeed observe nonspecific editing of our GFP reporter, especially for the cytidine base editors (additional file 1 FigS4a) as well as editing of Cas12a-ABE at off-target sites on subgenome B and D (additional file 1 FigS6c-f).*

Reviewer #2:

The manuscript of Gaillochet et al. describes the experiments to systematically optimize base editors for use in plants which the authors refer to as ITER. ITER is based on the transfection of 96-welled arrayed wheat and Corn protoplast. Every round of optimization can be achieved within three weeks which allows at fast pace enzyme optimization, which in combination with the high through-put approach is exceptional for plants.

The aim of the study was to optimize Cas12a based base editors. As a matter of fact, Cas12 base editor are less efficient than Cas9 base editors as they are based on a nuclease-dead and not on nickase-proficient DNA binding moiety. The authors tested different components of the enzyme for improvements namely the kind of editor, the crRNA, linker, NLS and mutations in LbCas12. The importance of all these factors was known form a row of published studies.

The authors succeeded in improving Cas12a-editors significantly from back ground noise to up to 20% editing at genomic sites in the best case. They also were able to document mutation induction in regenerated T0 plants at rates from 3 to almost 50 %

This is definitely an interesting piece of work. However; I want to raise two concerns:

What I missed was the documentation that the induced mutations were indeed transferred as expected to the T1 generation

*Response: A similar comment was raised by Reviewer 1. Briefly, as suggested, we have analyzed the inheritance of base edits in the T1 generation. We confirm that heterozygous and homozygous alleles are inherited in 22/24 lines tested (n=94 plants). We quantified the number of alleles in Fig. 4g and added our raw genotyping data in Additional file 2, Fig4g.*

What I found a worrisome ss that optimized editor induced beside the on-site mutation also quite efficiently off-site mutations at related sites. Have the author any evidence that the editor is not also inducing edits independent of the sequence in a genome wide manner?

*Response: We have observed non-specific base editing activity for the CBEs on the GFP reporter as well as activity of the Cas12a-ABE at off-targets (Fig. 4d; Additional file1 FigS6). These results are in line with published reports on off-target activity of base editors both on RNA and DNA (Jin et al 2019, Grunewald 2019). We agree that this is an important characteristic of the tools and have added a section at the end of the discussion to comment and suggest future improvements. We nevertheless feel that the genome-wide characterization of the Cas12a-ABEs is out of the scope of our present study and would warrant its own stand-alone study.*

Reviewer #3:

In the manuscript, the authors established an Iterative Testing of Editing Reagents (ITER) method to high throughput evaluate the editors' activity based on the visualization detection of corrected fluorescent proteins in 96-well arrayed protoplast system. Using this system, an LbCas12a-ABE were optimized to combine BP NLS, 6XGGGGS linker, ttLbCas12a, TadA9 and tRNA DR-DR crRNA element, and high base editing efficiencies were validated at endogenous sites and in transformed plants. Since the activity of current reported Cas12a-ABEs were quite limited, I believe the ITER-developed ABE is a good example to indicate the technical advances of the methods. Although it is interesting, I have some major concerns on the manuscript.

1. It is a well-used strategy to evaluate the editing frequency by examining the signals of corrected fluorescent proteins (e.g. Zong et al, 2022). The advantage of the ITER might be the reduced protoplast numbers and sensitive detection system. However, experimental proofs of comparing the ITER system with the established methods, such as flow cytometry analysis in a standard volume of protoplast, on genome editing tools are methodology required.

*Response: We would like to clarify that we make no claims comparing ITER to existing methodologies such as flow cytometry in the text. Our objective was to develop a high-throughput method to evaluate genome editing reagents and our data support the development of that tool (ITER), specifically for the development of base editors. The utility of ITER is the scale at which we can compare editing reagents by the use of automation. Data acquisition is essentially hands-free; the user has no additional work once the plate it loaded into the OPERA platform. We think the ITER strategy is a very powerful and easy-to-use tool. We added text in the discussion on lines 401-405 to elaborate on this point.*

*We are careful not to claim that ITER is superior than existing flow cytometry approaches. ITER is a tool, and like many tools, it may be more suited to certain questions or formats than others. This will only be determined with additional use. It is also not immediately obvious how to benchmark ITER vs. standard flow cytometry (or other) methods. There are a wide range of instruments and software available and any comparison would be limited to the instruments we have in our lab in addition to the genome editing technique being evaluated.*

The GFP and mCherry plasmids have transfection efficiencies of 40-65% in the system. However, the recently developed CRISPR tools, such as prime editors, are normally larger than 7kb in itself and might be increase continuously. I wonder the limited transfection frequency of big size plasmid would compromise the reliability of activity determination in the small well of the plate.

*Response: The largest plasmids we use contain the nuclease and range in size from 9.5-12.3 kb, with a mean of ~11kb. This is no different in size than the prime editing plasmids we currently use. If the transfection rate changes by using a different sized plasmid, or for any other technical reason, the analysis pipeline we have set up normalizes for transfection efficiency by detecting a constitutively-expressed mCherry-NLS (for high content imaging experiments) or a P2A-GFP-NLS tagging system (for sorting experiments). In either case, the user will be able to quantify the number of transformed cells and determine if the numbers are sufficient for the experiment and scale accordingly. We have added a sentence to the discussion illustrating this point:*

*“This also allows ITER to make comparisons between different vector systems that might have widely different transfection efficiencies due to plasmid size or other technical limitations.”*

2. The LbCas12a-ABEs were optimized by a single target in the ITER method, which might be affected by the context of the target site. Therefore, the robust of the system is not solid enough. I recommend to add at least another target in GFP in the assay.

*Response: We too think that assaying a single target can be problematic. This is why we re-tested a selection of base editors at endogenous gene targets both in protoplasts and stable wheat plants after testing them on the GFP reporter (Fig 2, Fig. 3 and Discussion line 405-414). We see the same stepwise improvements at the endogenous targets as observed on the GFP reporter. Therefore, we think the results are robust. While we cannot rule out that certain vector components might be more efficient at certain targets, overall we demonstrate a combination of Cas12a optimizations that work for multiple editing applications and target sites. We think this work provides researchers with a fairly efficient architecture to make additional improvements in the future.*

*We would also like to note that we did try two GFP reporters for Cas12a-ABE, but the first did not work (Additional file 1: Fig. S4a, b). While another GFP target site could likely be designed (requires a non-synonymous mutations to introduce a Cas12a PAM), we feel that testing the tools on endogenous sites is more relevant to test and confirm activity.*

And why compare the v9h and v11h in stable transformed plants. In the results of ITER, were found the efficiency of the two tools is somehow comparable. Why not detect the v12h and other low efficient versions in stable transformation to confirm the transient data? Since the wheat transformation may take long time, I recommend the examine the representative constructs in rice to fully validate the optimization.

*Response: As Reviewer 3 mentions, wheat transformation is a lengthy process with limited throughput. Because of time constraints in the project, we initiated stable wheat transformation at the time when v9h and v11h were two of the best architectures identified. Our analysis of these two versions in stable wheat plants support our findings from the ITER platform (v11 is generally better than v9) and demonstrate that we can efficiently optimize genome editing tools using this pipeline and obtain functional vectors for in vivo plant editing. While making a full comparison of the different Cas12a-ABE versions in stable plants of rice, wheat, or any other species would be very interesting, we feel it is out of scope for this report.*

3. It is interesting that the TadA9 significantly have significantly higher activity than TadA8e in the LbCas12a-fusions. However, nSpCas9-TadA9 has similar editing capability with the TadA8e variants at most targets (Yan et al, 2021). Can you discuss the potential reason?

*Response: It depends where you draw the error bars, but in Fig. 1d, Fig. 2a,c and Fig. 3a,c of Yan et al 2021., the authors show that TadA9 is equal to TadA8e at 5/9 targets and TadA9 is higher at 4/9 targets (none are lower). In our GFP optimization campaign (comparing v9 and v11), we see a significantly higher signal at the GFP target for v11 and in the stable plants, v11 is significantly more active at both target genes. However in the protoplast work in maize and wheat, the differences are not as clear. Based on the fairly limited number of targets we are looking at (9 in both studies), we think it is safest to say that the results of Yan et al are in line with our observations (Fig2, Fig.3).*

**Second round of review**

**Reviewer 1**

All my concerns had been well addressed in revised manuscripts. Therefore, I suggest this manuscript could be acceptable for publication on Genome Biology in the present form.

**Reviewer 2**

The authors addressed my two concerns in a satisfactory way.

**Reviewer 3**

I have no further comment.
